# Unique pattern of histogenesis of the parakeratinized epithelium on lingual prominence in the domestic goose embryos (*Anser anser f. domestica*)

**DOI:** 10.1038/s41598-021-02020-9

**Published:** 2021-11-23

**Authors:** Kinga Skieresz-Szewczyk, Hanna Jackowiak, Marlena Ratajczak

**Affiliations:** 1 Department of Histology and Embryology, Poznan University of Life Sciences, Wojska Polskiego 71C, 60-625 Poznan, Poland; 2 Laboratory of Electron and Confocal Microscopy, The Adam Mickiewicz University of Poznan, Umultowska 89, 61-614 Poznan, Poland

**Keywords:** Developmental biology, Embryology, Structural biology, Electron microscopy

## Abstract

A triangular lingual prominence (LP) is a characteristic part of the tongue in Anseriformes containing adipose tissue. The parakeratinized epithelium (PEp) covers the LP. Studies aimed to describe the histogenesis of PEp during the process of the intensive formation of the LP in domestic goose during embryonic period and to determine the structural readiness to perform a protective function. The study were conducted by using LM, SEM and TEM technique. The results revealed that on day 16th the undifferentiated epithelium of LP transformed into the typical avian multilayered epithelium. Contrary to pattern of histogenesis of parakeratinized epithelium on the lingual body, on the medial and lateral areas of the elongating and bulging LP were formed epithelial furrows. Which around 20th day, on lateral areas of LP deepened up to half of epithelium, whereas on the medial area began to fade. The ultrastructure of cells lying in furrows indicated progressive apoptosis-like degeneration. On the 25th day, shallow furrows were only present on lateral areas, where bulging of LP was continued. Whereas the epithelium on medial area started cornification by the accumulation of cytokeratin fibers. Lack of the periderm during the development of the PEp of the LP indicated its endodermal origin.

## Introduction

A characteristic feature of the mucosa of the avian tongue is that it is covered with two types of cornified epithelium, i.e., orthokeratinized and parakeratinized epithelium^1–6^. The area of occurrence of both types of cornified epithelia depends on the way of food intake and mechanism of food transport^2–6^.

The orthokeratinized epithelium is present at places directly involved in food intake. Namely, it forms the lingual nail involved in the pecking. It covers the conical papillae present on the lateral edges of the tongue in Anseriformes, involved in grazing green parts of plants^2–5,7^. It also covers the conical papillae present at the border between the lingual body and root, which move food into the oesophagus and prevent it from falling out of the beak cavity^2–5,7^.

In contrast, the parakeratinized epithelium covers the lingual mucosa over the dorsal surface of the lingual apex and body, where the food is transported into the oesophagus^1–5^. In Anserinae, due to the so-called over-tongue transport, the parakeratinized epithelium also covers the elevated posterior part of the body called the lingual prominence^4,8–12^.

The structure of the cornified layer is the main feature that differentiates the two types of cornified epithelia. In the orthokeratinized epithelium, the cornified layer is composed of keratinocytes lacking cell nuclei^4,5^. Whereas, in parakeratinized epithelium, keratinocytes in the cornified layer have cell nuclei with highly condensed chromatin^4,5^. At the same time, it should be emphasized that detailed ultrastructural observations have shown that the cornified layer in the ortho- and parakeratinized epithelium is composed of electron dense cells covered by electron lucent cells^6^. The cytoplasm of electron dense cells in the orthokeratinized epithelium is filled with bundles of keratin fibres arranged horizontally to the basal membrane^6^. In the parakeratinized epithelium, the bundles of keratin fibres cross each other^6^. In the case of electron lucent cells, it has been shown that in both ortho- and parakeratinized epithelium, the cell cytoplasm is filled with short and loosely arranged bundles of keratin fibres^6^.

The cornification process in birds involves accumulation in the keratinocytes of the specific beta-keratin, called Corneous Beta Proteins (CBP), and alpha-keratin^13–16^. Genes encoding the beta-keratin in birds, as in mammals, are placed in the Epidermal Differentiation Complex locus (EDC)^17^.

Recent molecular studies revealed that two types of cytokeratins, namely the alpha- and beta-keratin, that build bundles of keratin fibres, are present in both types of cornified epithelia^18,19^. However, alpha-keratin is generally found in the ortho- and parakeratinized epithelium, and beta-keratin is found primarily in the cornified layer, where the percentage of this cytokeratin was determined to be 70% in the orthokeratinized epithelium and 61% in the parakeratinized epithelium^18,19^.

The lingual prominence, mentioned previously is a raised posterior part of the body of the tongue typical for Anseriformes and in the Anserinae, like in the domestic goose, is covered with the parakeratinized epithelium^4,20–22^. The lingual prominence is triangular. Its base facing the posterior part of the tongue and has conical papillae. A median sulcus is present in the medial part of the lingual prominence. The microscopic investigation in the domestic goose revealed also the interesting features of the lingual prominence that its interior is built with yellow adipose tissue covered by lingual mucosa^4^. Generally, the lingual prominence is considered to be a ‘fat cushion’ that absorbs the pressures generated during food intake and food transport when the raised lingual prominence is pressed against the palate^4,8–12^. Interestingly, the pressure on the surface of the lingual prominence may stimulate the lingual glands, present on its sides, to release mucous^4^.

Own preliminary investigations on tongue development in the domestic goose indicate that lingual prominence starts to develop around 9/10 day of incubation in the form of an oval protrusion with a shallow longitudinal recess in the middle part^23^. Around day 15 of incubation, the protrusion start to elongate and bulge so that it takes a triangular shape with a median sulcus, as in the adult^23^.

The histogenesis of the parakeratinized epithelium so far was only studied on the flat dorsal surface of the lingual body and three stages of development were distinguished, i.e., the embryonic stage, transformation stage, and pre-hatching stage^24^. An essential finding of this study was that at the end of incubation, around the 23rd day, the parakeratinized epithelium has a fully developed cornified layer.

The aim of the current study is a microscopical and ultrastructural analysis of the development of the parakeratinized epithelium during the intensive growth of the lingual prominence in domestic goose between the 9th and 25th day of incubation. The obtained results will be used to determine the structural readiness of the parakeratinized epithelium on the lingual prominence to perform a protective function during the transport of food to the oesophagus. In addition, the performed observations will be used to conduct a comparative analysis of the development of the parakeratinized epithelium in two structurally different areas of the tongue, namely on the flat lingual body and raised lingual prominence.

## Results

### From 9th till 15th day of incubation (stage HH 32/33 to 39/40)

**Figure 1:**
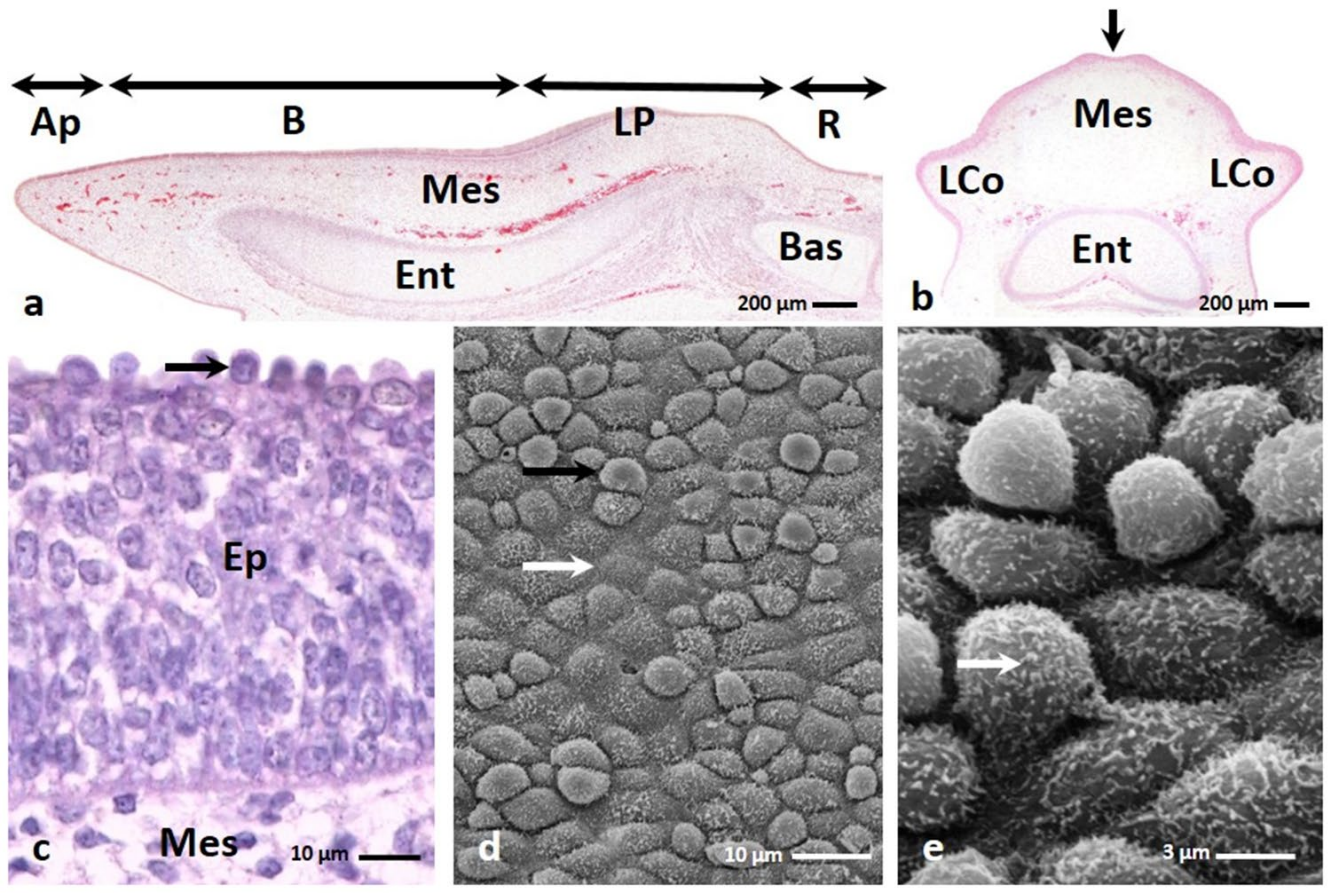
(**a**) 14th day of incubation. Cross section along the long axis of the tongue composing of the apex (Ap), body (B), raised lingual prominence (LP) and root of the tongue. Inside the tongue the primordium of the entoglossum cartilage (Ent) and basyhiale bone (Bas) are visible. Mes-mesenchyme. LM. Masson–Goldner staining. (**b**) 14th day of incubation. Transverse cross section of the lingual prominence with a shallow recess in the middle (arrow). On the side of the lingual prominence are visible primordia of the large conical papillae (LCo). Ent-entoglossum cartilage, Mes-mesenchyme. LM. Masson–Goldner staining. (**c**) 12th day of incubation. Cross section of the embryonic epithelium (Ep) on the lingual prominence. Violet colored cell nuclei of the superficial cells are present (arrow). Mes-mesenchyme. LM. Masson–Goldner staining. (**d**) 12th day of incubation. The surface of the lingual prominence epithelium with convex superficial cells (black arrow) and flat superficial cells (white arrow). SEM. (**e**) 12th day of incubation. Higher magnification of the surface of epithelial cells with numerous microvilli (arrow). SEM.

Between the 9th and 15th day of incubation, the oval raised lingual prominence was approximately 1.7 mm long (Fig. 1a). It consisted of the mesenchymal tissue, which undifferentiated, star-like shaped cells with round cell nuclei were loosely arranged (Figs. 1a, b, c). The Masson–Goldner staining revealed that the intercellular substance was stained light pink (Fig. 1c). Inside the tongue the primordia of the entoglossum cartilage and basihyale bone were present (Figs. 1a, b). In the middle part of the dorsal surface of the lingual prominence, the shallow recess was visible (Fig. 1b). On the side of the lingual prominence were visible primordia of the first large conical papillae (Fig. 1b).

The embryonic epithelium covering the lingual prominence comprised 11–12 layers of oval or rounded cells with a prominent cell nuclei and 1–2 nucleoli (Fig. 1c). The nuclei of the superficial cell dye in violet after Masson–Goldner staining (Fig. 1c). The observation of the epithelial surface in scanning electron microscopy revealed that superficial cells were round in shape, wherein the cells were equally convex and flat (Fig. 1d). On the cell surface were present single microvilli (Fig. 1e).

On the 9th day of incubation, the height of the epithelium was about 32.2 µm (Table 1). On the 10th day, an increase in the epithelial height to an average value of 60.7 µm was noted (Table 1). A decrease in epithelial height was observed between the 11th and 14th days of incubation. On the 15th day, the height of the epithelium increased to approx. 65.7 µm (Table 1). Statistical analysis showed that the difference in the height of the epithelium between the 9th and 10th day of incubation was statistically significant. On the remaining days, the differences in the height of the epithelium were not statistically significant.

**Table 1:** Results of measurements of the mean height of the epithelium of the lingual prominence in the domestic goose during embryonic development. X-mean value, min-minimum value, max-maximum value, SD-standard deviation.

Day of incubation	Height of the epithelium			SD
	X (µm)	Min (µm)	Max (µm)	
9	32.2	23.8	42.4	4.7
10	60.7	46.2	82.1	11.8
11	48.8	30.6	81.5	16.1
12	56.6	46.8	75.6	8.6
13	53.8	35.3	65.3	8.7
14	54.7	39.7	67.9	9.0
15	65.7	39.7	117.4	23.5
16	74.1	45.9	125.0	24.9
17	67.3	51.5	93.2	8.9
18	76.1	61.2	95.9	9.7
19	68.0	41.8	103.6	17.8
20	73.0	42.1	103.8	22.0
21	97.9	86.0	111.9	7.8
22	132.9	118.6	154.4	10.8
23	108.5	97.0	125.9	10.5
24	115.1	98.4	130.8	11.9
25	131.2	115.8	143.0	9.0

### Between 16th and 23rd day of incubation (stage HH 40 to 45)

**Figure 2:**
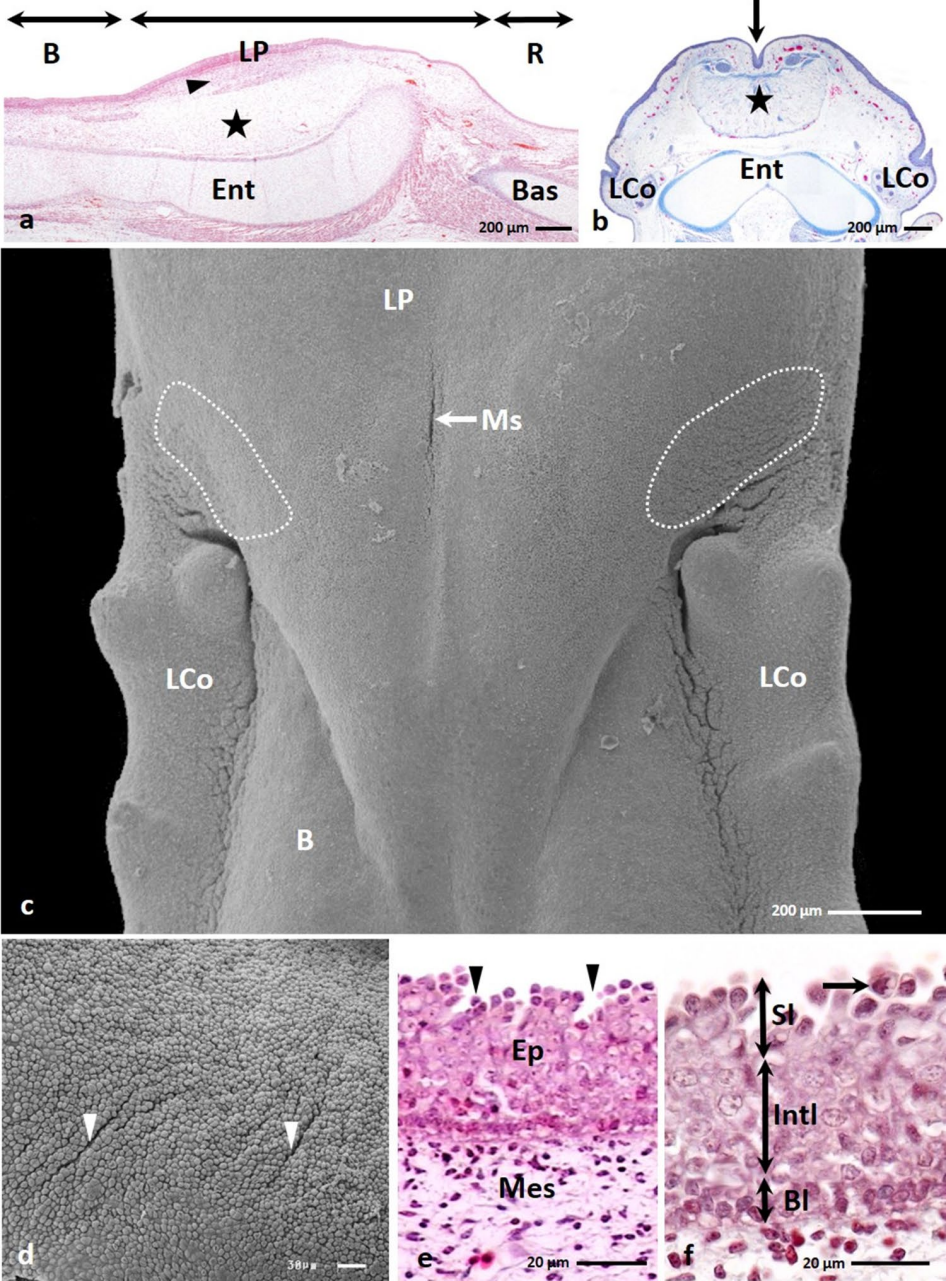
(**a**) 16th day of incubation. Cross section along the long axis of the caudal part of the body (B), lingual prominence (LP) and root of the tongue (R). Mesenchymal cells underneath the epithelium are densely arranged (arrow). Pre-adipocytes are present above the entoglossum cartilage (Ent) (asterisk). Bas-basihyale bone. LM. Masson–Goldner staining. (**b**) 18th day of incubation. Transverse cross section of the lingual prominence with median sulcus (arrow). Pre-adipocytes form an oval band (asterisk) over the entoglossum cartilage (Ent). LCo-primordium of the large conical papillae. LM. Masson–Goldner staining. (**c**) 16th day of incubation. View of the dorsal surface of the triangular lingual prominence (LP). On the lateral areas of the lingual prominence are present oval in shape areas with superficial furrows (dotted lines). B-body of the tongue, LCo-primordium of the large conical papillae. SEM. (**d**) 16th day of incubation. Magnification of the lateral area of the lingual prominence with epithelial furrows arrange in parallel rows (arrow heads). SEM. (**e**) 16th day of incubation. Cross section of the epithelium (Ep) of the lingual prominence with furrows (arrow heads). Mes-mesynchyme. LM. Masson–Goldner staining. (**f**) 16th day of incubation. Higher magnification of the epithelium on the lingual prominence consists of the basal (Bl), intermediate (Intl) and superficial layer (Sl). The cell nuclei in the superficial cells have condensed chromatin and are irregular in shape (arrow). LM. Masson–Goldner staining.

From 16th to the 19th day of incubation in the triangular lingual prominence, the area of mesenchymal tissue expanded, and the lingual prominence was bulging dorsally. Figure 2b,c presented a prominent median sulcus on the dorsal surface of the lingual prominence, which is about 257.6 µm long and reached a depth of about 158.7 µm.

The mesenchymal cells under the epithelium formed a compact arrangement (Fig. 2a). While the mesenchymal cells, located deeper, were the pre-adipocytes, which occupied the entire region of the lingual prominence reaching the surface of the entoglossum cartilage (Fig. 2a). The area with the immature adipose tissue in transverse cross section was oval in outline (Fig. 2b).

In the epithelium on the lateral areas of the lingual prominence were seen furrows, which covered an oval-shaped region at the anterior edge of the lingual prominence and extended to the primordium of the first large conical papilla of the lingual body (Fig. 2c). The region with epithelial furrows was about 0.19 mm^2^. The furrows were arranged parallel to each other, and their average length and depth was 83 µm and 13.7 µm, respectively (Figs. 2d).

On the 16th day of incubation, the epithelium covering the mucosa of the lingual prominence was built of 14–15 cell layers (Fig. 2e). In the epithelium, three epithelial layers: the basal, intermediate, and superficial layer were distinguished (Fig. 2f).

**Figure 3:**
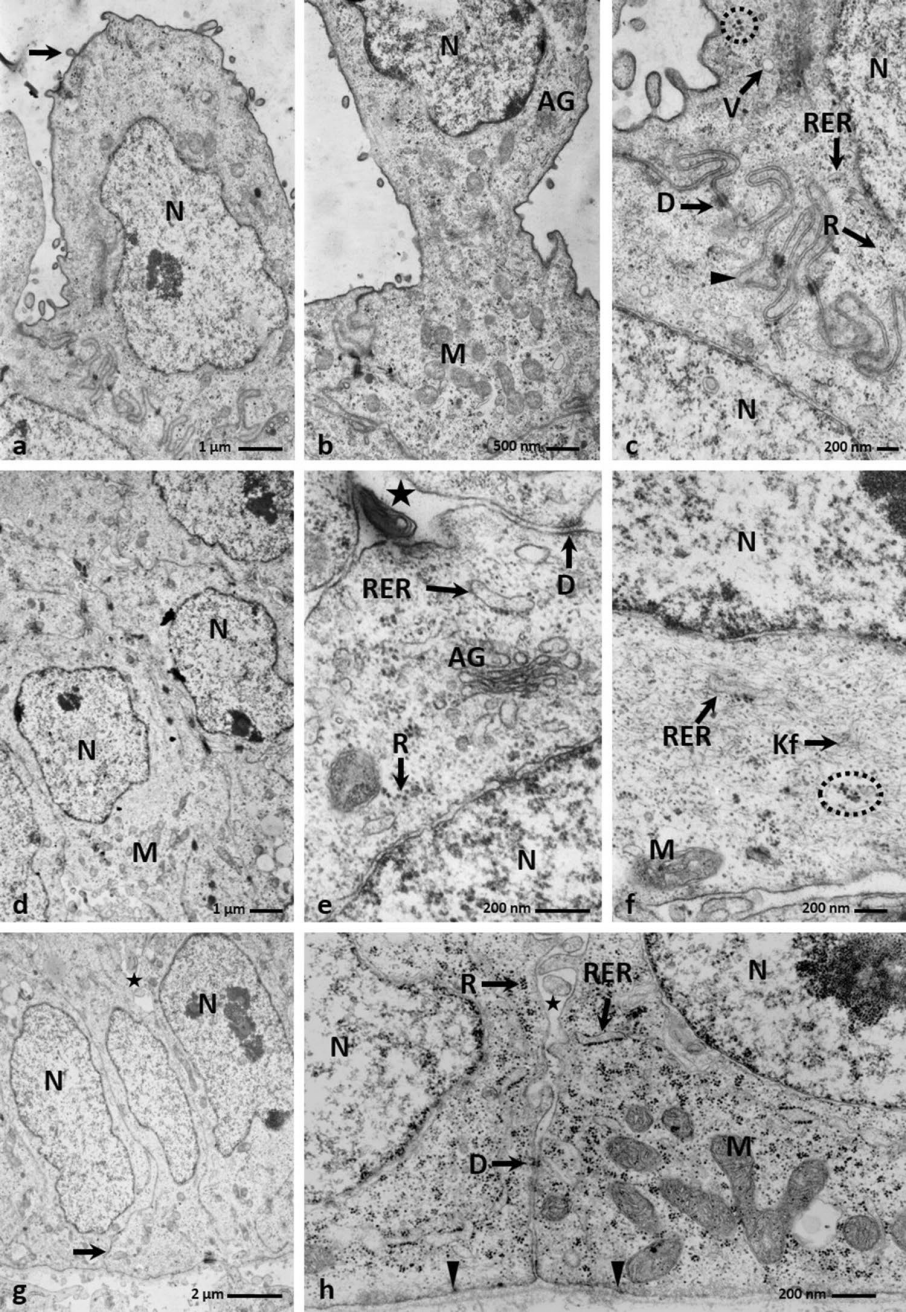
(**a**) 16th day of incubation. The convex cell of the superficial layer with sparse microvilli (arrow) and big cell nucleus (N) and few organelles. TEM. (**b**) 16th day of incubation. The convex cell of the superficial layer with the narrow base. In the basal part, are visible numerous mitochondria (M) and single Golgi Apparatus (AG). N-cell nucleus. TEM. (**c**) 16th day of incubation. The cytoplasm of the cell in the superficial layer with free ribosomes (R), rough endoplasmatic reticulum (RER), aggregates of glycogen (dotted line) and electron lucent vesicles (V). The cell membrane form mutual invagination (arrow head) and are connected by desmosomes (D). TEM. (**d**) 16th day of incubation. Micrograph of the round cells with rounded cell nucleus (N) in the intermediate layer. M-mitochondrium. TEM. (**e**) 16th day of incubation. The cytoplasm of the cell in the intermediate layer with rough endoplasmatic reticulum (RER), single Golgi Apparatus (AG) and free ribosomes in group (R). Between cells are present intercellular spaces (asterisk). Cell membrane are connected by desmosomes (D). N-cell nucleus. TEM. (**f**) 16th day of incubation. The cell cytoplasm of the intermediate layer with mitochondrium (M), rough endoplasmatic reticulum (RER), thin bundles of keratin fibers (Kf) and aggregates of glycogen (dotted line). N-cell nucleus. TEM. (**g**) 16th day of incubation. Micrograph of the cylindrical cells in the basal layer. Cell nucleus (N) are oval in shape. In the apical part are visible intercellular spaces (asterisk). In the basal part the cell membrane are closely adjacent to each other (arrow). N-cell nucleus. TEM. (**h**) 16th day of incubation. The cell cytoplasm of the basal layer with numerous mitochondria (M), rough endoplasmatic reticulum (RER), single Golgi Apparatus (AG) and ribosomes arrange in groups (R). Cells are attached to the basal membrane by hemidesmosomes (arrow heads). Between cell are present intercellular spaces (asterisk) and cell membranes are connected by desmosomes (D). N-cell nucleus. TEM.

The observation proceeding in the light microscopy and transmission electron microscopy revealed that the cells in the basal layer were cylindrical and had oval cell nuclei with or without nucleoli (Figs. 2f, 3g). In the cell cytoplasm of the basal part of the cell were present numerous mitochondria, rough endoplasmatic reticulum, single cisterns of Golgi Apparatus, and free ribosomes arranged in groups of 4–5 ribosomes (Fig. 3h). The cell membranes of the neighbour cells, just above the basal membrane, were closely adjacent to each other and connected by single desmosomes (Figs. 3g, h). In the apical part, between the basal cells were present intercellular spaces (Figs. 3g, h). The basal cells were attached to the basal membrane with well–formed hemidesmosomes (Fig. 3h).

The intermediate layer consisted of the round cells with lightly coloured cell cytoplasm and rounded cell nuclei (Figs. 2f, 3d). In the cell cytoplasm were observed numerous mitochondria, free ribosomes arranged in groups of 4–5 ribosomes, single Golgi Apparatus, and cisterns of rough endoplasmatic reticulum (Figs. 3d, e, f). Figure 3f presented thin bundles of keratin fibres and glycogen grains. Between neighbouring cells were intercellular spaces, and cell membranes were connected by desmosomes (Fig. 3e).

Light microscopy and transmission electron microscopy observations revealed that cells on the epithelial surface and in the furrows were round and convex, and had few microvilli on the surface (Figs. 2f, 3a). Their cell nuclei were strongly stained with hematoxylin and had 1–2 nucleoli (Figs. 2f, 3a). In some superficial cells, the cell nuclei had an irregular shape, and their chromatin was condensed (Fig. 2f). Many superficial cells were highly elongated and had a narrowed basal part of the cell forming the stalk (Fig. 3b). In the cell cytoplasm were many mitochondria, single Golgi Apparatus, rough endoplasmatic reticulum and glycogen grains (Figs. 3b, c). In the cell cytoplasm were also free ribosomes and single electron lucent vesicles (Fig. 3c). The cell membrane of the superficial cells adhered to the membranes of underlying cells, and mutual invaginations of these cell membranes were visible (Fig. 3c). The cell membrane were connected by single desmosomes (Fig. 3c).

On day 16 of incubation, the epithelial height increased and reached an average of  74.1 μm (Table 1). Statistical analysis showed that the difference in epithelial height between days 15 and 16 was statistically significant.

**Figure 4:**
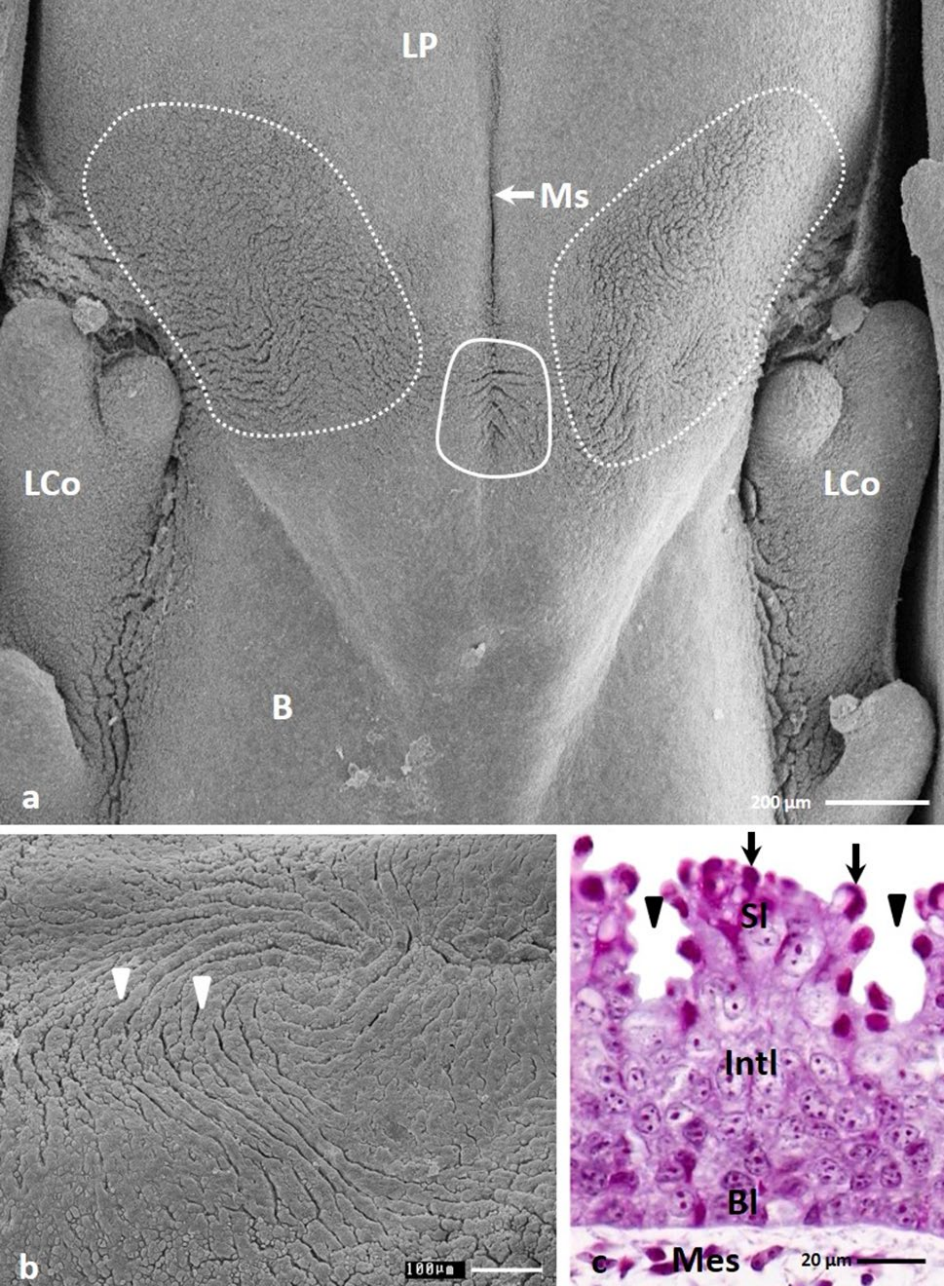
(**a**) 17th day of incubation. View on the surface of the lingual prominence with parallel arranged furrows (continuous line) on the sides of the anterior part of the median sulcus (Ms). The lateral areas with epithelial furrows (dotted lines) extend from the anterior edges towards the medial part of the lingual prominence (LP). B-body of the tongue, LCo-large conical papillae. SEM. (**b**) 17th day of incubation. Higher magnification of the lateral area of the lingual prominence with wavy arrange furrows (arrow heads). SEM. (**c**) 18th day of incubation. Cross section of the epithelium of the lingual prominence with superficial furrows (arrow heads). The arrows show the condensed cell nuclei in cells of the superficial layer. Bl-basal layer, Int-intermediate layer, Mes-mesenchyme, Sl-superficial layer. LM. Masson–Goldner staining.

From the 17th till the 19th day of incubation, the area with superficial epithelial furrows on the lateral areas of the lingual prominence extended from the anterior edges towards the medial part of the lingual prominence (Fig. 4a). It occupied a region of approximately 0.66 mm^2^. On these days, superficial furrows were also present on the medial area of the lingual prominence, on the sides of the anterior part of the median sulcus, where they occupied a region of approximately 0.03 mm^2^ (Fig. 4a). The arrangement of the furrows on the lateral areas of the lingual prominence was wavy (Figs. 4a, b). In contrast, on the medial area parallel furrows were located perpendicularly to the median sulcus (Fig. 4a).

The structure of the epithelium on the 17th day did not change compared to the previously examined days. In the following days, i.e., days 18 and 19 of incubation, light microscopy observations of epithelium showed that all cell nuclei of the superficial cells had condensed chromatin (Fig. 4c).

The mean epithelial height increased to 76.1 μm on the 18th day of incubation (Table 1). No significant statistical differences in epithelial height were noted between the 17th and 19th day of incubation.

**Figure 5:**
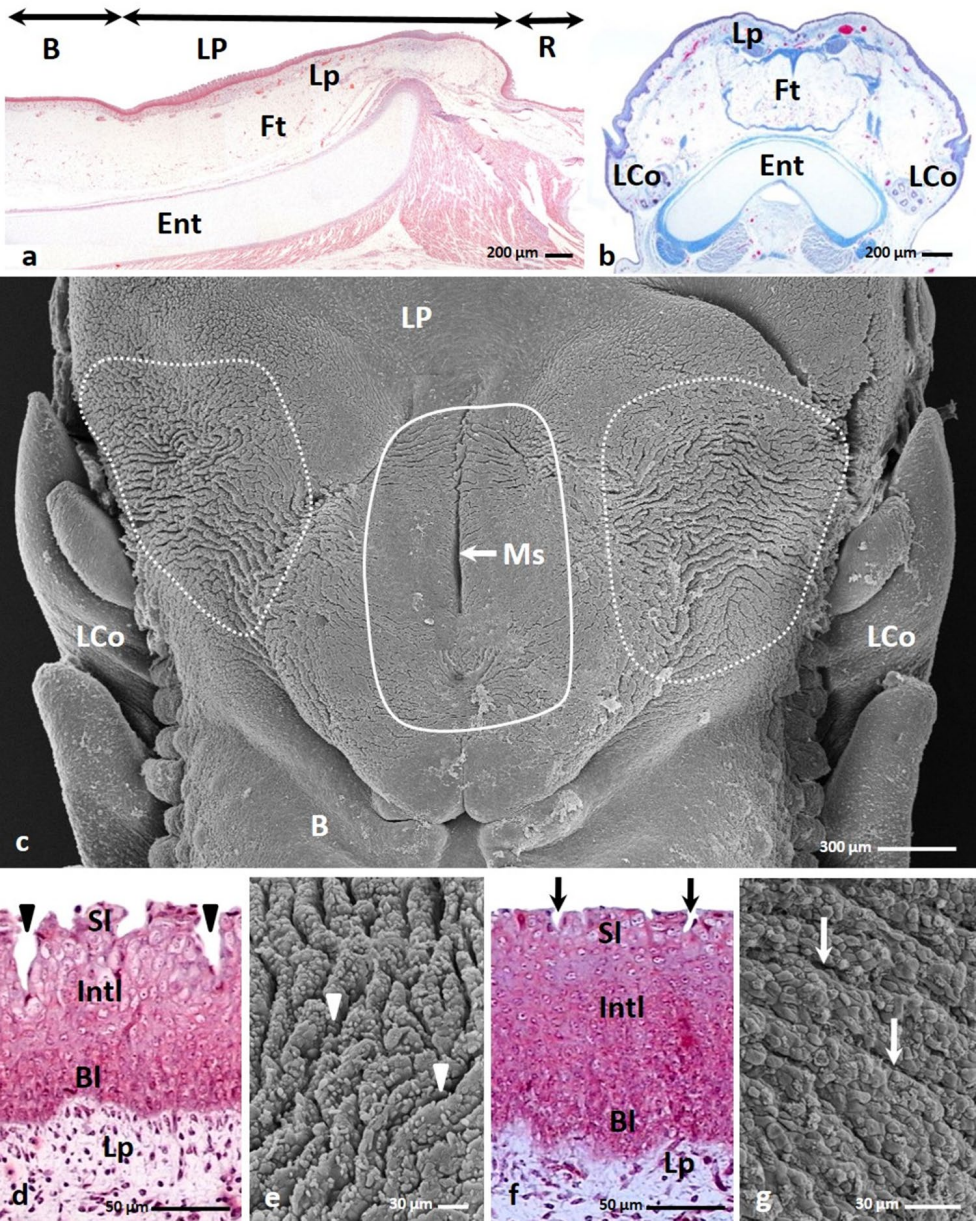
(**a**) 21st day of incubation. Cross section along the long axis of the caudal part of the body (B), lingual prominence (LP) and root of the tongue (R). The lingual prominence consisted of the lamina propria (Lp) and fat tissue (Ft). Ent-entoglossum cartilage. LM. Masson–Goldner staining (**b**) 21st day of incubation. Transverse cross section of the lingual prominence with a fat tissue (Ft) surrounded by thick bundles of collagen fibres. Ent-entoglossum cartilage, Lp-lamina propria, LCo-large conical papillae. LM. Masson–Goldner staining (**c**) 21st day of incubation. View on the dorsal surface of the lingual prominence (LP) with lateral areas (dotted line) and medial area (continuous line) raise above the flat surface of the lingual body (B). The epithelial furrows on the medial area are located along the entire length of the median sulcus (Ms). LCo-large conical papillae. SEM (**d**) 22nd day of incubation. Cross section of the epithelium of the lateral area of the lingual prominence with distinct furrows (arrow heads). Bl-basal layer, Int-intermediate layer, Lp-lamina propria, Sl-superficial layer. LM. Masson–Goldner staining (**e**) 21st day of incubation. Higher magnification of the lateral area of the lingual prominence with distinct furrows (arrowheads). SEM. (**f**) 22nd day of incubation. Cross section of the epithelium of the medial area of the lingual prominence with shallow furrows (arrows). The polygonal cells in the intermediate layer (Intl) have round or oval cell nuclei. Cells in the superficial layer (Sl) have condensed cell nuclei. Bl-basal layer, Lp-lamina propria. LM. Masson–Goldner staining (**g**) 21st day of incubation. Higher magnification of the medial area of the lingual prominence with shallow furrows (arrows). The superficial cells are convex. SEM.

Between the 20th and 23rd day of incubation, the lateral areas and the medial area of the lingual prominence raised above the flat surface of the lingual body (Fig. 5c). The region of superficial furrows on the medial area extended along the entire length of the median sulcus and occupied approximately 1.1 mm^2^ (Fig. 5c). The region occupied by the furrows on the lateral areas of the lingual prominence was approx. 2.16 mm^2^.

The cross sections of the lingual prominence at the 21st and 22nd day of incubation showed bundles of collagen fibres in the lamina propria of the mucosa (Figs. 5a, b, d, f). Just below the lamina propria of the mucosa, fat tissue surrounded by thick bundles of collagen fibres forming a kind of envelope was present (Figs. 5a, b). The area formed by the fat tissue was symmetrical concerning the surface of the lingual prominence and the median sulcus.

Observation of the epithelial surfaces of the lingual prominences in the scanning electron microscopy and their histological sections in the light microscopy showed that the epithelial furrows on the medial area of the lingual prominence were shallow, reaching a depth of ca. 11.5 µm (Figs. 5f, g). In contrast, the furrows on the lateral areas are deeper and have a ca. 39.6 µm in depth (Figs. 5d, e).

The epithelium on the medial area of the lingual prominence was composed of 20–21 cell layers (Fig. 5f). The cells in the basal layer of the epithelium did not change their structure compared to the previous studied days. In contrast, cells in the intermediate layer were polygonal in shape and had round or oval cell nuclei with one or two nucleoli (Fig. 5f). Cells on the surface and in the furrows were round and had condensed cell nuclei (Fig. 5f). Observation of the epithelium by scanning electron microscopy showed that the superficial cells were slightly convex (Fig. 5g).

The epithelium on the lateral areas of the lingual prominence was composed of 15–16 cell layers (Fig. 5d). The cell shapes and the staining of the cell cytoplasm of the basal layer were unchanged from the previously examined days.

**Figure 6:**
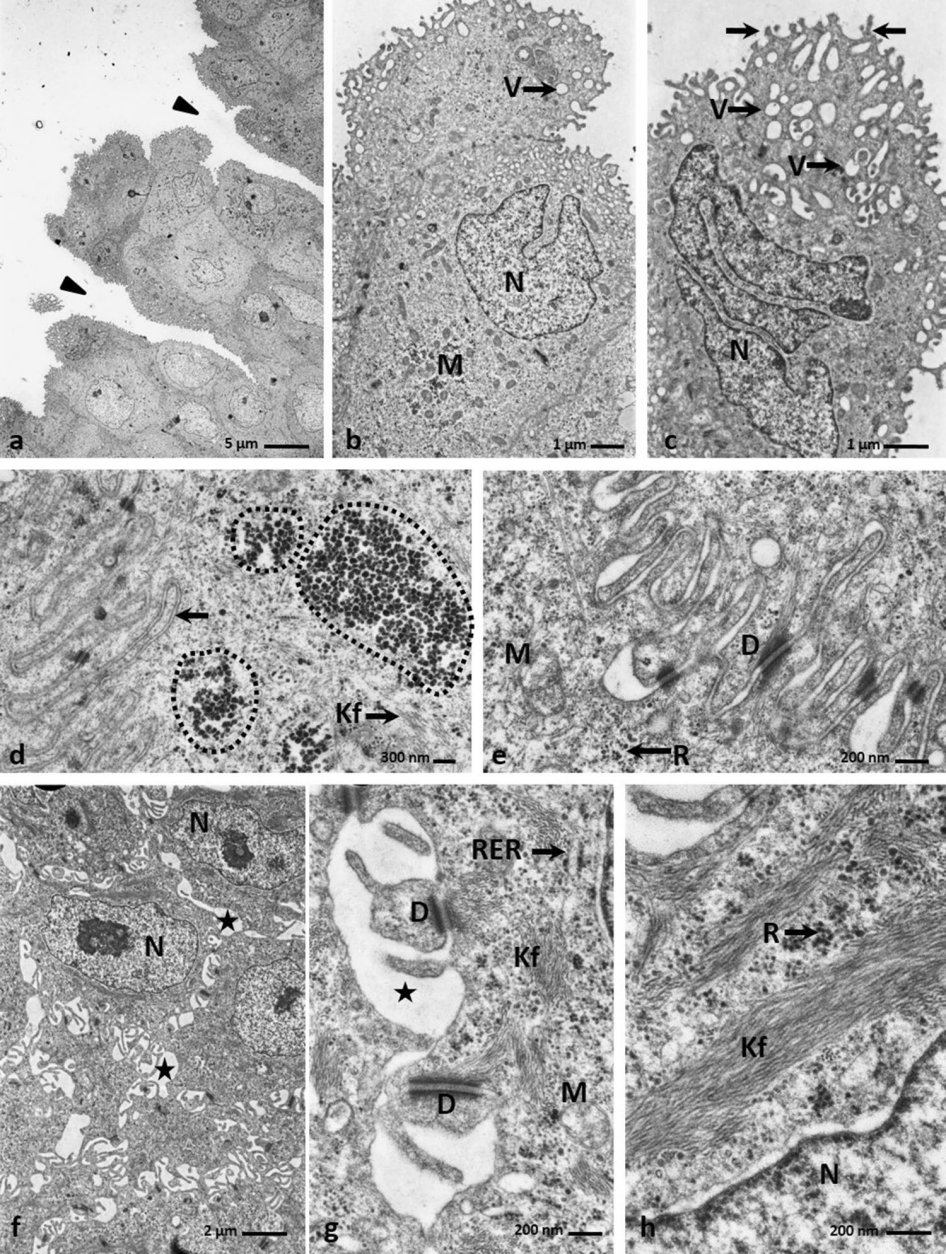
(**a**) 23rd day of incubation. Micrograph of the superficial layer of the epithelium on the lateral area of the lingual prominence with distinct furrows (arrow heads).TEM. (**b**) 23rd day of incubation. The cell of the superficial layer, which cell nucleus (N) has an inward nuclear membrane. In the cell cytoplasm are present numerous mitochondria (M) and electron lucent vesicles (V). TEM. (**c**) 23rd day of incubation. The cell of the superficial layer with fragmented cell nucleus (N) and numerous vesicles (V). On the cell surface are present microvilli (arrows). TEM. (**d**) 23rd day of incubation. The cytoplasm of the cell of superficial layer with cell membrane invagination of the adherent cells (arrow). In the cytoplasm are present the aggregation of glycogen (dotted liens) and bundles of keratin fibers (Kf). TEM. (**e**) 23rd day of incubation. Higher magnification of the invaginations of cell membranes of the superficial layer connected by numerous desmosomes (D). M-mitochondrium, R-ribosomes. TEM. (**f**) 23rd day of incubation. Micrograph of the cells in the intermediate layer of the epithelium on the lateral area of the lingual prominence with numerous and large intercellular spaces (asterisks). N-cell nucleus. TEM. (**g**) 23rd day of incubation. The cytoplasm of the cell in the intermediate layer with single mitochondria (M), rough endoplasmatic reticulum (RER) and keratin fibers (Kf). Between neighboring cells are many intercellular spaces (asterisk) and cell membrane were connected by desmosomes (D). TEM. (**h**) 23rd day of incubation. The cell cytoplasm of the intermediate layer with the keratin fibers (Kf) arrange along the cell nucleus (N) and free ribosomes (R). TEM.

The cells of the intermediate layer were polygonal in shape, and their oval cell nuclei had single nucleoli (Figs. 5d, 6f). Ultrastructural observations of the intermediate layer showed numerous and large intercellular spaces between adjacent cells and cell membranes were connected by desmosomes (Figs. 6f, g). There were numerous thick bundles of keratin filaments in the cell cytoplasm arranged along the nuclear membrane (Figs. 6g, h). In the cytoplasm, free ribosomes were arranged in groups of 3–4 ribosomes, single mitochondria, and an rough endoplasmatic reticulum (Figs. 6g, h).

Observations of the superficial layer indicated that the cells were convex and round in outline, and the cell nuclei had condensed chromatin (Figs. 5d, 6a). In some cells, the cytoplasm around the nuclei, after Masson—Goldner staining, was brightly stained and vacuolated (Fig. 5d). The cell nuclei had a variable outline, either because they were round with a single nucleolus or had an irregular shape with an inward nuclear membrane (Fig. 6b). Some cell nuclei were even fragmented (Fig. 6c). In the cell cytoplasm of the superficial cells, there were many mitochondria, free ribosomes, clusters of glycogen grains, thin bundles of keratin fibers, and numerous electron lucent small vesicles (Figs. 6b, c, d, e). The cell membranes of neighbouring cells closely adhered to each other and mutually inserted themselves (Fig. 6d). Desmosome were present between the cell membranes (Fig. 6e). The apical membrane of the cell formed numerous microvilli (Fig. 6c).

On the 22nd day, the height of the epithelium increased and reached an average of 132.9 μm (Table 1). On the 23rd day, the epithelial height decreased to an average value of approx. 108,5 μm (Table 1). The difference in epithelial height between days 21, 22, and 23 was statistically significant.

### 24th and 25th day of incubation (stage HH 45 to 46)

**Figure 7:**
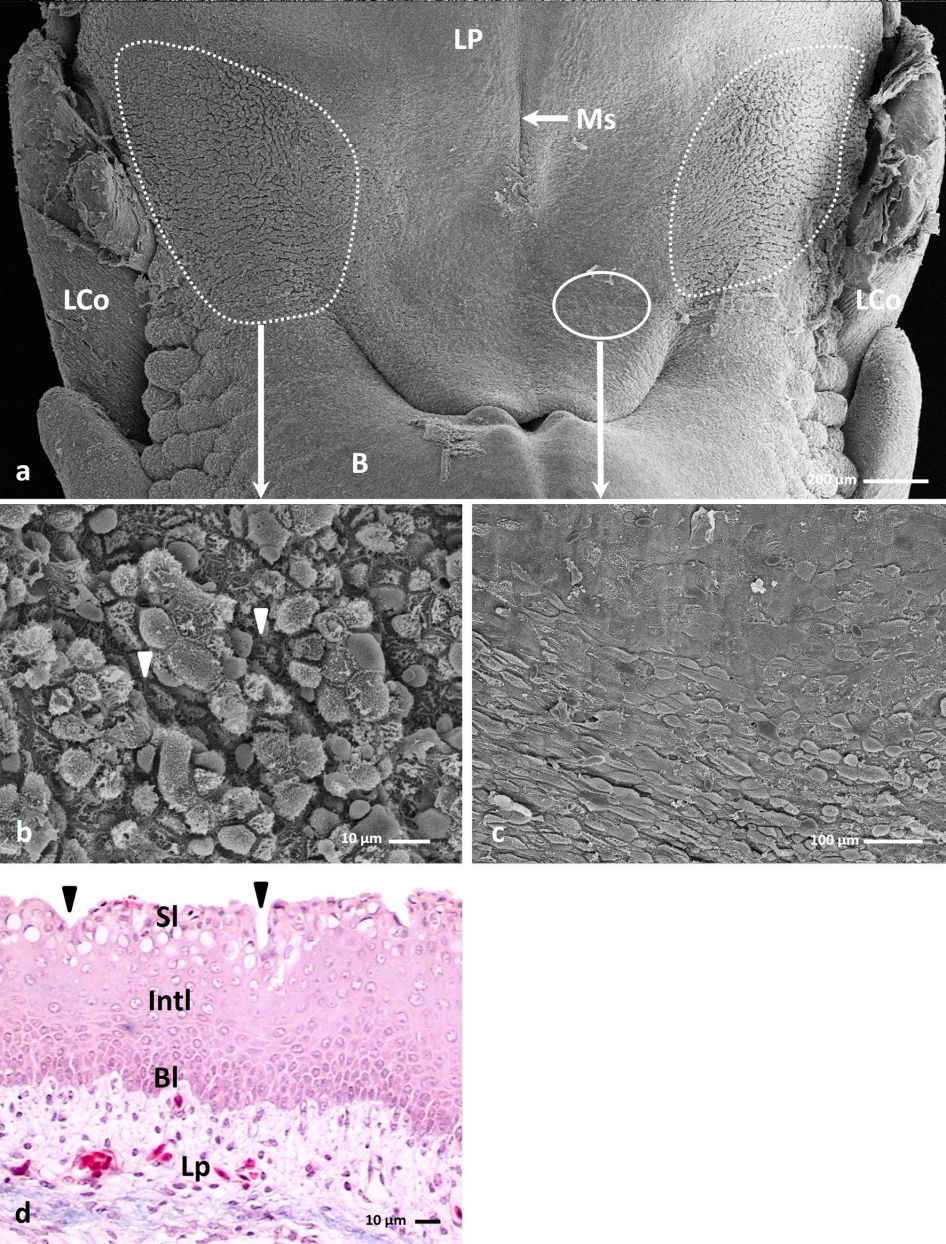
(**a**) 24th day of incubation. View on the dorsal surface of the lingual prominence with flat medial area (circle) and shallow furrows on the lateral areas (dotted line). B-body of the tongue, LP-lingual prominence, Ms-median sulcus, LCo-large conical papillae. SEM. (**b**) 24th day of incubation. Higher magnification of the surface of the epithelium on the lateral area of the lingual prominence with shallow furrows arrange perpendicular to the long axis of the tongue (arrowheads). SEM. (**c**) 24th day of incubation. Higher magnification of the surface of the epithelium on the flat medial area of the lingual prominence without furrows. SEM. (**d**) 24th day of incubation. Cross section of the epithelium of the lateral area of the lingual prominence with shallow furrows (arrow heads). Bl-basal layer, Int-intermediate layer, Lp-lamina propria, Sl-superficial layer. LM. Masson–Goldner staining.

On these days, the flat medial area of the lingual prominence was smooth and devoid of furrows (Figs. 7a, c). On the raised lateral areas of the lingual prominence, shallow, superficial furrows were present, which were arranged perpendicular to the long axis of the tongue and occupied a region of approximately 0.87 mm^2^ (Figs. 7a, b, d). The average depth of furrows was approximately 17.0 µm.

**Figure 8:**
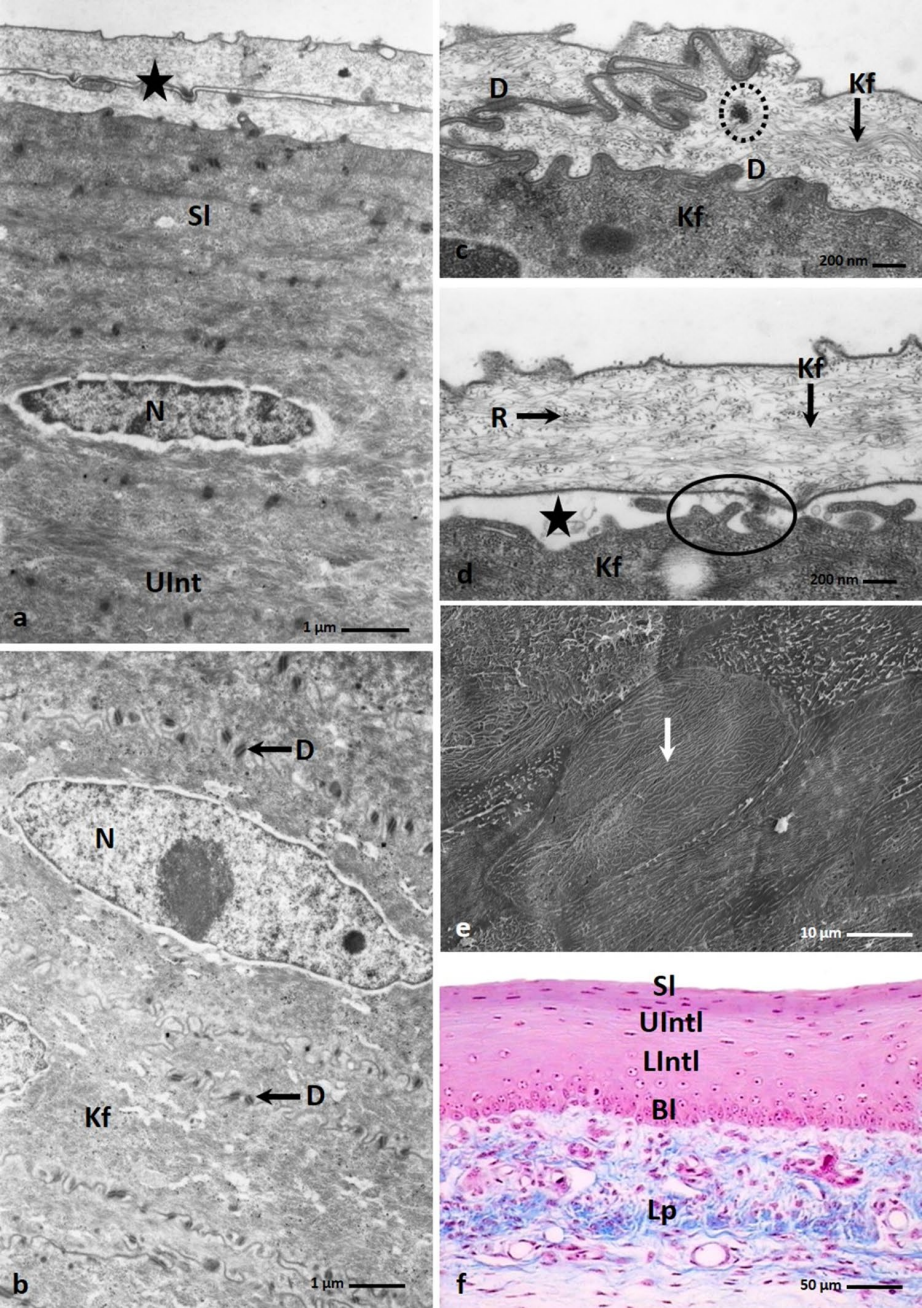
(**a**) 25th day of incubation. Micrograph of the superficial layer (Sl) and upper part of the intermediate layer (UInt) of the epithelium on the medial area of the lingual prominence. The superficial layer is composed of the electron lucent cells (asterisk) and electron dense cell located below. N-cell nucleus. TEM. (**b**) 25th day of incubation. Micrograph of the cells of the lower part of the intermediate layer. Cell cytoplasm contains numerous bundles of the keratin fibers (Kf). Cell membranes are connected by desmosomes (D) on short cytoplasm projections. N-cell nucleus. TEM. (**c**) 25th day of incubation. Higher magnification of the cells of the superficial layer which cell membrane are closely arranged and connected by desmosomes (D). In the cell cytoplasm the aggregation of the glycogen (dotted line) is present. Kf-keratin fibers. TEM. (**d**) 25th day of incubation. The cell cytoplasm of the electron lucent cell of the superficial layer of the epithelium filled with thin bundles of the keratin fibers (Kf) loosely arranged and free ribosomes (R). The cell cytoplasm of the electron dense cell is wholly filled with bundles of keratin bundles (Kf) arranged horizontally. Between electron lucent and electron dense cell are present the intercellular spaces (asterisk). The short invagination of the cell membranes form a microprojections (oval mark). TEM. (**e**) 25th day of incubation. Higher magnification of the surface of the superficial epithelial cells with parallel arranged microprojections (arrow). SEM. (**f**) 25th day of incubation. Cross section of the epithelium on the flat medial area of the lingual prominence consisted of the basal layer (Bl), lower part of the intermediate layer (LInt), upper part of the intermediate layer (UInt) and superficial layer (Sl). Lp-lamina propria. LM. Masson–Goldner staining.

On the 25th day of incubation, the epithelium of the medial area of the lingual prominence was composed of 20–21 cell layers (Fig. 8f). Changes in the structure of the intermediate layer and the superficial layer could be observed. In the intermediate layer, the shapes of cell nuclei changed and the two parts of the intermediate layer were distinguished. In the lower part, the cell nuclei were oval, and in the upper part, they were flat (Fig. 8f). After Masson—Goldner staining, the cell cytoplasm of both parts of the intermediate layer had a light pink colour (Fig. 8f). Transmission electron microscope observations of the cell cytoplasm in the intermediate layer indicated numerous bundles of keratin fibres that almost filled the cell (Fig. 8b). Small intercellular spaces were visible between adjacent cells, and the cell membranes were connected by numerous desmosomes (Fig. 8b).

Cells in the superficial layer were flattened, and their cytoplasm was pink coloured (Fig. 8f). Some superficial cells had strongly flattened cell nuclei (Fig. 8f). Transmission electron microscopy studies indicated that the superficial layer was composed of 3–4 layers of electron dense cells covered by two layers of electron lucent cells (Fig. 8a). The electron dense cells had strongly flattened cell nuclei, and the cytoplasm was wholly filled with bundles of keratin fibres arranged horizontally (Fig. 8a). There were no cell nuclei in electron lucent cells, and the cytoplasm was filled with thin, single keratin fibres that were loosely arranged (Figs. 8c, d). The cell membranes of some electron lucent and electron dense cells created mutual invaginations and were connected through numerous desmosomes (Fig. 8c). Some of the electron lucent cells were peeling off, and an intercellular space was visible between the cells (Figs. 8d). In Fig. 8d, we observed that previous invaginations of cell membranes were now microprojections. Only single free ribosomes and glycogen grains were present in the cell cytoplasm of the superficial cells (Figs. 8c, d). Observation of the cell surface in the scanning electron microscopy showed that the microprojections were aligned parallel to each other (Fig. 8e).

The average height of the epithelium on the 24th day of incubation is 115.1 µm and on the 25th day was 131.2 µm (Table 1). The difference in epithelial height between the 24th and 25th day of development was statistically significant.

## Discussion

Literature published to date about histogenesis of the lingual epithelium in avian species is limited. The only studies concerning the development of the orthokeratinized epithelium on the conical papillae of the tongue were proceeded in chicken by using the scanning electron microscopy^30^ and in the domestic goose and duck by using light microscopy and scanning electron microscopy^23,25,31^. Later histological and ultrastructural studies in the scanning and transmission electron microscopy were conducted by the Skieresz-Szewczyk et al.^24,26^ and describe the development of the orthokeratinized epithelium on the ventral surface of the lingual apex and parakeratinized epithelium of the flat lingual body in the domestic duck. Those studies give detailed information about the histodifferentiation of these epithelia during embryonic period.

Present histological and ultrastructural observations have allow to characterize a not-yet-described process of histodifferentiation of the embryonic epithelium covering the growing lingual prominence in the domestic goose into parakeratinized epithelium.

As mentioned in the introduction, the development of the lingual prominence of the domestic goose occurs around day 10 of incubation and continues until hatching^23^. These observations indicate that one of the first features of the lingual prominence is the formation of a median sulcus on its convex surface and its successive elongation up to the hatching. The turning point in the development of the lingual prominence is day 15 of incubation when the lingual prominence takes on a triangular shape elongated towards the lingual body^23^. It is worth mentioning that the process of tongue elongation is accompanied by the gradual development of successive pairs of conical papillae, starting from the lateral edges of the anterior part of the lingual prominence towards the apex of the tongue, whose development starts from the 11th day of incubation^25^. The formation of the primordia of the first pair of large conical papillae, which are directly adjacent to the anterior edges of the lateral areas of the lingual prominence, is fascinating. On day 12 of development, these primordia divide into two equal-sized parts, which by day 14/15 change in size so that the medial part is smaller than the lateral part^25^. These studies have also shown that there is a continuous growth of lingual prominence throughout embryonic development. Around day 25 of incubation, it becomes both two times longer and wider.

In the present microscopic study, the transformation of the lingual prominence in the domestic goose were observed. By day 16 of incubation, the interior of the raised lingual prominence was filled with mesenchymal tissue, extending as far as the entoglossum cartilage. Mesenchymal tissue formed the primordium of a structure called the 'fat cushion' of lingual prominence in adult birds of Anserinae. After the 16th day of incubation, when the lingual prominence was already triangular, the mesenchymal cells differentiated into fibroblasts and pre-adipocytes so that by the 20th/21st day of incubation, the interior of the prominence was filled with fat tissue surrounded by a sheath made up of bundles of collagen fibres.

Previous histological and ultrastructural studies on the development of the parakeratinized epithelium of the flat lingual body and the orthokeratinized epithelium of the ventral surface of the lingual apex in domestic duck have shown that differentiation of the cornified epithelia occurs at three developmental stages: embryonic stage, transformation stage and pre-hatching stage^24,26^.

The present results of the histodifferentiation of the parakeratinized epithelium of the lingual prominence in the domestic goose had distinguished the following developmental stages.

The embryonic stage, characterized by undifferentiated epithelial cells convex over the surface of the epithelium, on the lingual prominence in domestic goose, lasted until day 15 of incubation. While in the parakeratinized epithelium of the lingual body and the orthokeratinized epithelium, the embryonic stage lasts shorter until 13th/14th day of incubation^24,26^.

The next developmental stage, the transformation stage, in the epithelium of the lingual prominence in the domestic goose lasted from 16th to 23rd day of incubation. This stage in the parakeratinized epithelium of the lingual body lasts from 15th to 22nd day of incubation, and in the orthokeratinized epithelium from 14 to 20th day^24,26^.

The transformation stage is characterized by the formation of the three epithelial layers typical of the adult (basal, intermediate, and superficial layer), the division of the intermediate epithelial layer into two structurally distinct parts (lower and upper part), and the presence of osmophilic mesh-like periderm granules^24,26^.

During the development of the parakeratinized epithelium of the lingual prominence in domestic goose, a three-layered structure of the lingual prominence epithelium was observed from day 16 of incubation. However, no differentiation of the intermediate layer into two parts or formation of periderm granules was recorded.

The periderm granules have been observed during the development of the epidermis of the skin and its cornified appendages in reptiles and birds^13,16,27^. It has been determined that the presence of periderm granules during the development of the epidermis and cornified epithelia of the avian tongue indicates the ectodermal origin of these epithelia^24,26^. The absence of periderm granules during the development of the parakeratinized epithelium of the lingual prominence might indicated that this epithelium originated from the endoderm. The results of the current study showed agreement with embryological statements that the epithelia of the posterior third of the tongue derived from the endoderm^28,29^.

The phenomenon of the transformation stage of the parakeratinized epithelium of the lingual prominence in domestic goose was the presence of unique furrows in the superficial layer of the epithelium. The superficial furrows are not observed during the development of the parakeratinized epithelium on the lingual body^24^. Initially, superficial furrows were noted at the anterior edges on the lateral areas of the lingual prominence on day 16 of incubation and one day later, also on its medial area. According to Skieresz-Szewczyk et al.^23^, on the 16th day, the lingual prominence changes its shape from oval to triangular due to elongation. Till the 22nd day, the length and width of lingual prominence increase^23^. These morphological changes could cause that the area occupied by the superficial furrows increased, on the lateral areas 3-times and on the medial area 36-times. Additionally, around the 20th/21st day of incubation, the narrow medial part of the lingual prominence widens^23^. At that time, the superficial furrows in the epithelium of the medial area became two times shallower and started to disappear. On the other hand, on the lateral areas, the furrows became almost two times deeper, which might be related to raising of the anterior edges of the lateral areas of the lingual prominence.

Analyzing the ultrastructure of the parakeratinized epithelium of the lingual prominence at the transformation stage, the structure of the cells forming the superficial layer in the area of epithelial furrows drew attention. These cells were strongly convex on the surface of the epithelium and sometimes fused with epithelial cells located below only by a narrow stalk. In these cells, initially, there was a folding of the nuclear membrane, followed by fragmentation of the cell nucleus and the presence of numerous vesicles. These features might be evidence of progressive degeneration of these cells by apoptosis. However, it should be emphasized that throughout the entire transformation stage, the convex superficial cells were closely connected to the cells situated underneath by desmosomes.

Both at the embryonic and the transformation stage, the superficial epithelial cells had few microvilli, which is a feature observed during the development of the parakeratinized epithelium of the lingual body and the orthokeratinized epithelium in the domestic duck and during tongue development in the chicken^23–26,30^. The presence of microvilli on the cell surface, in our opinion, could be considered as a typical feature for undifferentiated cells and cells during transformation.

The third stage of epithelial development, the pre-hatching stage, in the parakeratinized epithelium of the lingual prominence in the domestic goose covered days 24 and 25 of embryonic development.

In the parakeratinized epithelium of the lingual body, this stage starts from day 23 and in the orthokeratinized epithelium from day 21 and continues until day 25 of incubation^24,26^. The pre-hatching stage is the stage during which the cornified layer of the epithelium is formed.

The present study did not show a typical cornified layer in the epithelium of the lingual prominence. However, division of the intermediate layer into two parts, i.e., a lower part and an upper part, was noted.

The prehatching stage of the parakeratinized epithelium of the lingual prominence in domestic goose was characterized by the complete disappearance of the superficial furrows in the epithelium on the medial area. The furrows at the lateral areas were shallowed. The complete disappearance of the furrows on the lateral areas of the lingual prominence probably occurred after hatching and might be related to the further growth of the lingual prominence.

In the pre-hatching stage, the superficial layer of the parakeratinized epithelium of the lingual prominence was made up of two ultrastructurally different cell types, i.e., electron lucent cells lacking cell nuclei and undergoing exfoliation and electron dense cells having cell nuclei with condensed chromatin and cytoplasm filled with bundles of keratin fibres. Further differentiation of the superficial layer into a cornified layer occurred after hatching and was stimulated by mechanical pressure associated with the initiation of feeding.

Superficial cells at the pre-hatching stage had microprojections on the apical surface. Such structures are described as microridges and are considered a typical feature of mature keratinocytes^32,33^. A study by Skieresz-Szewczyk et al.^24^ shows that microprojections/microridges are present at sites corresponding to the presence of desmosomes. The results of the current study confirmed this information.

A specific feature of the superficial cells of the epithelium of the lingual prominence in domestic goose at the transformation stage and the pre-hatching stage was the presence of clusters of glycogen granules in the cell cytoplasm. Previous studies on the development of the parakeratinized epithelium on the lingual body and the orthokeratinized epithelium have shown differences in the distribution of glycogen^24,26^. In orthokeratinized epithelium, glycogen granules are observed at the embryonic stage in the cell cytoplasm of superficial cells^26^. In the parakeratinized epithelium of the lingual body, glycogen granules are accumulated during the embryonic stage in both superficial and intermediate cells and the transformation stage in the cell cytoplasm of the basal layer^24^. The presence of glycogen during the development of the parakeratinized epithelium of the lingual body and the orthokeratinized epithelium is related to the need for storage material necessary for the morphological transformation of epithelial cells. In the case of the parakeratinized epithelium of the lingual prominence, glycogen granules, both at the transformation and pre-hatching stages, might indicate the need to synthesize cytokeratins and the formation of keratin fibres after hatching and thus the formation of the cornified layer.

The results of the morphometric analysis of the epithelial height on the lingual prominence showed that there was a two-times increase in epithelial height during the embryonic stage. In the transformation stage, fluctuations in epithelial height ranging from about 70 µm to about 130 µm were observed associated with the intensive development of superficial epithelial furrows. Finally, the height of the epithelium of the lingual prominence in the domestic goose increased four-times.

In conclusion, the histogenesis of the parakeratinized epithelium on the surface of the lingual prominence in goose occurred during three developmental stages. In our opinion, the phenomenon of formation and regression of unique superficial epithelial furrows were related to the intensive growth of the lingual prominence and histogenesis of the yellow adipose tissue. The growth of the lingual prominence and the formation of yellow adipose tissue caused mechanical forces that stress the subepithelial connective tissue and the differentiating epithelium. It could cause cell proliferation in the basal layer of the epithelium, cell apoptosis, and furrow formation. The fact that no periderm with periderm granules was formed during the development of the parakeratinized epithelium of the lingual prominence indicated an endodermal origin compared to the previously found ectodermal origin of the parakeratinized epithelium of the lingual body. Just before hatching, the ‘fat cushion’ of the lingual prominence in the domestic goose was fully developed. However, the cornified layer, typical for adults, was missing, and its further development would take place after hatching.

The unique pattern of the histogenesis of the parakeratinized epithelium related to the growth of the lingual prominence in the domestic goose might be a phenomenon in other avian species belonging to the Anseriformes and having lingual prominence. This issue requires further investigation.

## Material and methods

Incubation of the 74 fertilized eggs of the domestic goose and removing the embryos from eggs were proceeded according to protocol by Skieresz-Szewczyk et al.^23^. Taking into account own observations of domestic goose embryos and on the studies of Hamburger and Hamilton^34^ and Koecke^35^, Hamburger-Hamilton (HH) developmental stages are assigned to the particular days of incubation.

The study was conducted on tongues between the 9th and 25th day of incubation. The collected tongues were examined using LM, SEM, and TEM techniques. Four tongues were collected every 24 h and subsequently fixed in 10% formalin. After 48 h of fixation, every two tongues were prepared for light microscopic observations (LM), and two other tongues were prepared for examination under a scanning electron microscope (SEM). Tissue samples for the light microscopy study were dehydrated in a series of increasing ethanol concentrations (70–96%) and routinely embedded in Paraplast® (Sigma-Aldrich, Germany). Paraplast blocks were cut into sections of 4.5–5 μm. Histological sections were stained using the Masson–Goldner trichrome staining technique^36^ and were examined using an Axioscope2plus light microscope (Zeiss, Germany).

The 3–6 measurements of the height of the epithelium from ten histological sections were proceeded to determine in total 30–60 measurements. Histological measurements were made using the Multiscan computer morphometric system (ver. 10.2, CSS, Warsaw, Poland) and were statistically analysed using Statistica (ver. 12.5; StatSoft, Poland). Statistical analyses covered the following parameters: the mean value (X) with standard deviation (SD), the minimum value (min), and the maximum value (max). The t-test for independent samples (level of significance α = 0.05) was used to determine the statistical significance of differences in mean values of the height of the epithelium between the particular days of incubation.

Tissue samples for the scanning electron microscopy study were dehydrated in a series of increasing ethanol concentrations (70–99.8%) for 15 min, then in a mixture of 96% ethanol and acetone (proportion 1:1) for 10 min and acetone for 5 min (Skieresz-Szewczyk et al.^24,26^). The tissue samples were then dried at the critical point using CO_2_ (Critical Point Dryer K850, EMITECH). All samples were mounted on aluminium stubs covered with carbon tabs, sputtered with gold with 10 μm (Sputter Coater S 150B, EDWARDS), and observed under the SEM LEO 435 VP microscope (ZEISS) at an accelerating voltage of 10–15 kV min (Skieresz-Szewczyk et al.^24,26^).

Tissue samples for the transmission electron microscopy were proceeding according to the protocol (Skieresz-Szewczyk et al.^24,26^). Two tongues from days 16, 23, and 25 were fixed in a 2.5% solution of glutaraldehyde (Serva Electrophoresis, Germany) in cacodylate buffer (pH = 7.2) (Sigma-Aldrich, Germany) (Skieresz-Szewczyk et al.^24,26^). They then were fixed in a 4% osmium tetroxide (Sigma-Aldrich, Germany) solution in cacodylate buffer (pH = 7.2) at a temperature of 0–4 °C min. Fixed tissue samples were dehydrated in a series of increasing ethanol concentrations (30–96%), a mixture of ethanol and propylene oxide (proportion 1:1), and pure propylene oxide at a temperature of 0–4 °C. Samples were embedded in Epon 812 epoxy resin (Serva Electrophoresis, Germany) with 2% DMP-30 (Serva Electrophoresis, Germany). Polymerization of the resin was run for three days at a temperature of 37 °C, 45 °C, and 60 °C. The sections of 70 nm were counterstained with 2% aqueous uranyl acetate and lead citrate, and observations and the photographic documentation were taken under a JEM1200 EX II transmission electron microscope (JEOL Co.).

The present study, according to Polish law and EU directive no. 2010/63/EU does not require approval of the Local Ethical Committee for Experiments on Animals in Poznan.

All methods were carried out in accordance with relevant guidelines and regulations. The study was carried out in compliance with the ARRIVE guidelines.

## Abbreviations

AG: Golgi Apparatus
Ap: Apex of the tongue
B: Body of the tongue
Bas: Basihyale bone
Bl: Basal layer
D: Desmosome
Ent: Entoglossum cartilage
Ep: Epithelium
Ft: Fat tissue
Int: Intermediate layer
LP: Lingual prominence
Kf: Keratin fibers
LCo: Large conical papillae
LInt: Lower part of the intermediate layer
Lp: Lamina propria
M: Mitochondrium
Mes: Mesenchyme
Ms: Median sulcus
N: Cell nucleus
R: Root of the tongue
R: Ribosomes
RER: Rough endoplasmatic reticulum
Sl: Superficial layer
UInt: Upper part of the intermediate layer
V: Vesicle
